# Let’s Live Healthier: The Relationship between Suicidal Behavior and Physical Activity in an Age-, Gender-, and Body Mass Index-Matched Adults

**DOI:** 10.3390/ijerph17228350

**Published:** 2020-11-11

**Authors:** Jeong-Hui Park, Myong-Won Seo, Hyun Chul Jung, Jung-Min Lee

**Affiliations:** 1 Department of Physical Education, Kyung Hee University, 1732 Deokyoungdaero, Giheung-gu, Yongin-si, Gyeonggi-do 17014, Korea; jeonghee@khu.ac.kr; 2 Department of Taekwondo, Kyung Hee University, 1732 Deokyoungdaero, Giheung-gu Yongin-si, Gyeonggi-do 17014, Korea; myongwonseo@khu.ac.kr; 3 Department of Coaching, Kyung Hee University, 1732 Deokyoungdaero, Giheung-gu, Yongin-si, Gyeonggi-do 17014, Korea; jhc@khu.ac.kr

**Keywords:** physical activity, suicidal behaviors, accelerometer, measurement, national survey

## Abstract

The purpose of this study was to identify the association between physical activity (PA) and predictors of suicidal behaviors and to investigate whether the different PA measurements influence the association between PA and suicidal behaviors in South Korean adults. This study analyzed data from the National Health and Nutrition Examination Survey 2014–2015 data. The study selected participants who checked suicide-related questions as “Yes” (*n* = 99) and checked suicide-related questions as “No” (*n* = 99) in the questionnaire. The age, gender, and body mass index of participants between the two groups were matched. The moderate to vigorous PA (*p* = 0.000) and sedentary PA (*p* = 0.000), measured by accelerometers, were a significant risk factor for suicidal behaviors. Furthermore, the number of steps was a considerable difference between the two groups (healthy group: 61,495.76 steps; suicide group: 40,517.34 steps), and the accelerometer and questionnaire also showed significant differences. The study demonstrated that there were significant associations with physical activity and socioeconomic status and suicidal behaviors in anthropometry (i.e., age, gender, height, weight) matched groups. Additionally, this study highlights the importance of the assessment of PAs, and increasing PA levels could reduce the incidence of suicidal behaviors.

## 1. Introduction

While the World Health Organization (WHO) has regulated that suicide is a critical public health problem, however, approximately 800,000 people worldwide die from suicide each year [1]. The number of people who attempt suicide is increasing every year, and this unfavorable trend has become the greatest public health concern in some countries such as South Korea. The average suicide rate among Korean is indicated at 24.6 per 100,000 people, the highest rate in the past decade among countries of the Organization for Economic Cooperation and Development (OECD) [2,3].

Many studies have shown that one preventive factor against suicide and suicide attempt is physical activity [4,5,6]. Regular physical activity (PA) has positive effects on general physical and psychological health such as stress, anxiety, and depression [7]. The study by John et al. demonstrated that participating in PAs could help to promote positive mental health and reduce depression in adults in the United Kingdom [8]. The study indicated that depression was affected by insufficient PA in the older age group (>65) rather than in the younger age group (<65). Additionally, Rothon et al. [9] reported that PA was closely related to depressive symptoms and suicidal behaviors. This study demonstrated that poor body image also contributed to depression when they perceived themselves as overweight, causing low self-esteem. Therefore, low PA levels might cause significant changes as high levels of depression and low self-esteem as well as being physically inactive have also been linked to increased risks for suicidal behaviors in adolescents [9].

Some studies have presented the opposite results of the relationships between suicide attempts and participation of PAs, even though suicide has a positive link to participating in PA. Based on the study by Thomas et al., while the intensity and frequency of PA did not significantly affect suicide behaviors in American people aged 13 to 34 years [10], another study indicated that more significant amounts of PAs and participating in more vigorous-intensity PAs helped adolescents to reduce suicidal behaviors [11]. Likewise, in Korea, Lee et al. indicated 40.64% of Korean adolescents who had suicidal thoughts over the past year participated in regular or frequent vigorous-intensity physical activities over moderate-intensity physical activity (13.31%) [12]. However, some studies found that adolescents who did not participate in physical activities were 1.42 times more likely to attempt suicide than those who participated in vigorous-intensity physical activities more than three times a week [13,14].

The different results from the earlier studies on the same population may be related to differences in applied PA assessment. Most studies typically measure PA through self-reported questionnaires for practical reasons [15]. However, physical activity data from a self-reported questionnaire are vulnerable to reporting bias [16,17,18]. According to the study by Troiano and colleagues, PA measured in self-reported methods indicated significantly higher estimates than those compared with objectively measured PA using accelerometers [19]. Lee et al. [12] also demonstrated that a low correlation (r = 0.26) was found between accelerometers and questionnaires to measure PA in Koreans aged 30 to 64.

Therefore, the first aim of this study was to investigate the effective methods for measuring PA and to identify the association between PA and suicide behaviors accurately. The second aim of this study was to compare the differences between objective (i.e., accelerometers) and subjective (i.e., questionnaire) methods in measuring the amount of physical activity between groups of participants who had attempted or thought of suicide and those who had not. Importantly, we matched age, gender, height, weight, waist circumference, and body mass index for both population groups. 

## 2. Materials and Methods

### 2.1. Design

The National Health and Nutrition Examination Survey (NHANES) is a series of cross-sectional population studies conducted by the Korea Centers for Disease Control and Prevention (KCDCP) to obtain information on health behavior, chronic diseases, prevalence, and food and nutrition of the representative Korean population through interviews, surveys, and physical examinations. PA was assessed using the survey and the accelerometers with the number of adults who agreed to participate in the sixth, second, and third year (2014–2015) of the National Health and Nutrition Survey. The current study was approved by the Institutional Review Board of the Korea Centers for Disease Control and Prevention (2013-12EXP-03-5C, 2015-01-02-6C).

### 2.2. Participants

The PA data using accelerometers consisted of 1768 persons, 977 in 2014, and 791 in 2015. However, one accelerometer data were partially missing and excluded (*n* = 1767). Additionally, data from persons (*n* = 342) who wore accelerometers for at least 10 h a day less than four days a week (*n* = 1425) were excluded for reliability purposes (*n* = 1425). It also excluded eight missing persons from the PA data in the questionnaire (*n* = 1417). Among those who surveyed in the questionnaire, the study selected participants who checked suicide-related questions as “Yes” (*n* = 99) and checked suicide-related questions as “No” and matched age, gender, and body mass index with those who checked “Yes” (*n* = 99). The sample size calculation assumed 80% power with alpha of 0.05 and was calculated using G-Power software version 3.1.9 [20] based on previous literature [21]. Adequate sample size was calculated to be at least 64 participants per group.

### 2.3. Accelerometer Physical Activity

Data for this study were obtained from the National Health and Nutrition Examination Survey (NHANES) conducted by the Korea Centers for Disease Control and Prevention (KCDCP) from 2014 to 2015. The data had Korean health information using accelerometers and were analyzed by SAS (SAS Institute, Inc., Cary, NC, USA) following analysis code. The accelerometers used GT3X+ of ActiGraph (ActiGraph, Pensacola, FL, USA) and the study participants wore accelerometers at the left (or right) waist for seven consecutive days, except for sleeping, showering, and swimming. The epoch of the accelerometer’s physical activity data was one minute. If the counts per minute (CPM) indicating the intensity of physical activity remained at 0 for more than 60 min, it was considered a non-wearing time (two minutes below CPM 100 were allowed). Accelerometer and physical activity intensities were classified by applying the cut-points of Troiano et al. (e.g., CPM of 2020 for moderate-intensity PA and CPM of greater than 5999 for vigorous-intensity PA). In addition, to obtain valid PA data, we examined the accelerometer data that included a minimum of 10 h per day and a minimum of 4 days per week [4]. 

### 2.4. Self-Report Physical Activity

The questionnaire used a Korean-language version of the Global Physical Activity Questionnaire (GPAQ) with proven reliability and validity. The GPAQ consists of questions about physical activity that are usually conducted for a week, and questions about moderate physical activity related to location movement are included. Participants should answer the average number of minutes of physical activity within the work and leisure categories for each moderate and vigorous-intensity physical activity. The questionnaire validated the data by applying the procedures presented in the Global Health Organization’s GPAQ Analysis Guideline. PA was calculated by “MET level × minutes × number of activities per week” for each intensity, with 8.0 METs for vigorous physical activities and 4.0 METs for moderate physical activities. As categorical variables, the physical activity levels of the participants were classified into low physical activity, moderate physical activity, and vigorous physical activity, respectively. Cronbach’s alpha in the current study was calculated as 0.070.

### 2.5. Suicidal Behaviors

Suicidal behaviors were investigated through questions on suicide ideations, suicide plans, and suicide attempts. The ideation of suicide asked, “Have you ever thought of attempting suicide once in the past year?”. The suicide plan asked, “Have you ever made any concrete plans to attempt suicide in the past year?”, and the suicide attempt asked, “Have you ever actually tried to suicide in the past year?”. The responses were also measured as “Yes” or “No”. Participants who answered “Yes” to any of the three questions were classified as a suicide group, and those who answered “No” to all three questions were classified as a health group.

### 2.6. Data Analysis

The demographic information of the participants was summarized by descriptive statistics in SPSS 25.0 version (SPSS Inc., Chicago, IL, USA). This was examined by the descriptive statistics of the participants who were divided into two groups (suicide and healthy group) and the participants’ personal information such as gender, age, education, occupation, and average monthly income. Additionally, the participants’ body composition (height, weight, waist circumference, and body mass index (BMI) were analyzed using the independent t-test for continuous variables. The *p*-value was utilized to demonstrate whether the correlation between the participants’ body composition and their reliability was examined by 95% confidence intervals for two groups in each variable. The independent t-test was also utilized to investigate the differences between the questionnaire-assessed PA and accelerometer-assessed PA, and *p*-value was used to evaluate whether there was a correlation between the two methods. Furthermore, multinomial logistic regressions were used to identify the predictors of suicidal behaviors in adults and to determine the differences between the risk factors indicated by different measurements. To demonstrate predictors related to suicidal behaviors, all variables (demographic information, body composition, and physical activity measured by two methods) were included in the multinomial logistic regression analysis as independent variables, and the suicidal and healthy group were used as a dependent variable. Results from this analysis are presented as odds ratios (OR) with 95% confidence intervals (95% CI) and statistical significance set by *p* < 0.05. All data processing and statistical analysis used SPSS 25.0.

## 3. Results

Table 1 summarizes the demographic participants’ characteristics of adults using descriptive statistics (*n* = 198). Demographic characteristics such as gender, age, education, average monthly income, and occupation of participants are presented in number and proportion in Table 1. Overall, the number of females was more than twice that of males, and the number of participants in their 50s and 20s was relatively higher. The mean ± SD was calculated as 162.12 ± 9.36 cm for height, 63.81 ± 12.60 kg for weight, 80.76 ± 10.65 cm for waist circumference, and 24.24 ± 4.12 kg·m^−2^ for BMI in the suicide group, and 161.94 ± 7.97 cm for height, 63.85 ± 11.72 kg for weight, 80.76 ± 10.29 cm for waist circumference, and 24.31 ± 3.82 kg·m^−2^ for BMI in the healthy group. The results indicated that there were no significant differences in height (*p* = 0.87), weight (*p* = 0.98), waist circumference (*p* = 0.92), and BMI (*p* = 0.89) in the two groups. The education level of the suicide group was the highest with 47.5% in high school graduates, followed by 25.3% with undergraduate graduates. However, that of the healthy group were undergraduate graduates with 41.4%, followed by high school graduates with 39.4%. Additionally, more than half of the participants earned less than 2000 thousand KRW (USD 1650), and the occupation of participants revealed the highest portion in unemployment, followed by service workers and shop sales workers, respectively, in the two groups.

Table 2 is the result of multinomial logistic regression analysis to demonstrate factors that can predict suicidal behaviors in adults and to measure suicide behavior predictors more accurately. According to Models 1 and 2 in Table 2, suicide behaviors were unrelated to gender, educational level, and occupation, however, the moderate to vigorous physical activity (MVPA) (OR = 0.995; CI = 0.992–0.998; *p* = 0.001) and sedentary physical activity (SPA) (OR = 1.001; CI = 1.000–1.001; *p* = 0.040) measured by accelerometers, compared to the MVPA (OR = 1.000; CI = 1.000–1.000; *p* = 0.682) and the SPA (OR = 1.000; CI = 1.000–1.000; *p* = 0.168) measured by questionnaires, were significant risk factors in predicting suicidal behaviors in adults. In addition, the results of Models 3 and 4 in Table 2 showed differences in whether some factors were related to suicidal behaviors or not, depending on the two different measurements. Therefore, the results proved that age, height, weight, and waist circumference are irrelevant factors for suicidal behaviors, and MVPA (OR = 0.995; CI = 0.993–0.998; *p* = 0.001) and SPA (OR = 1.001; CI = 1.000–1.001; *p* = 0.007), measured by accelerometers in Model 3, were significant risk factors in predicting suicidal behavior. On the other hand, MVPA (OR = 1.000; CI = 1.000–1.000; *p* = 0.655) and SPA (OR = 1.000; CI = 1.000–1.000; *p* = 0.194) measured by questionnaires in Model 4 were found to be unrelated to the factors for predicting suicidal behaviors.

**Figure 1:**
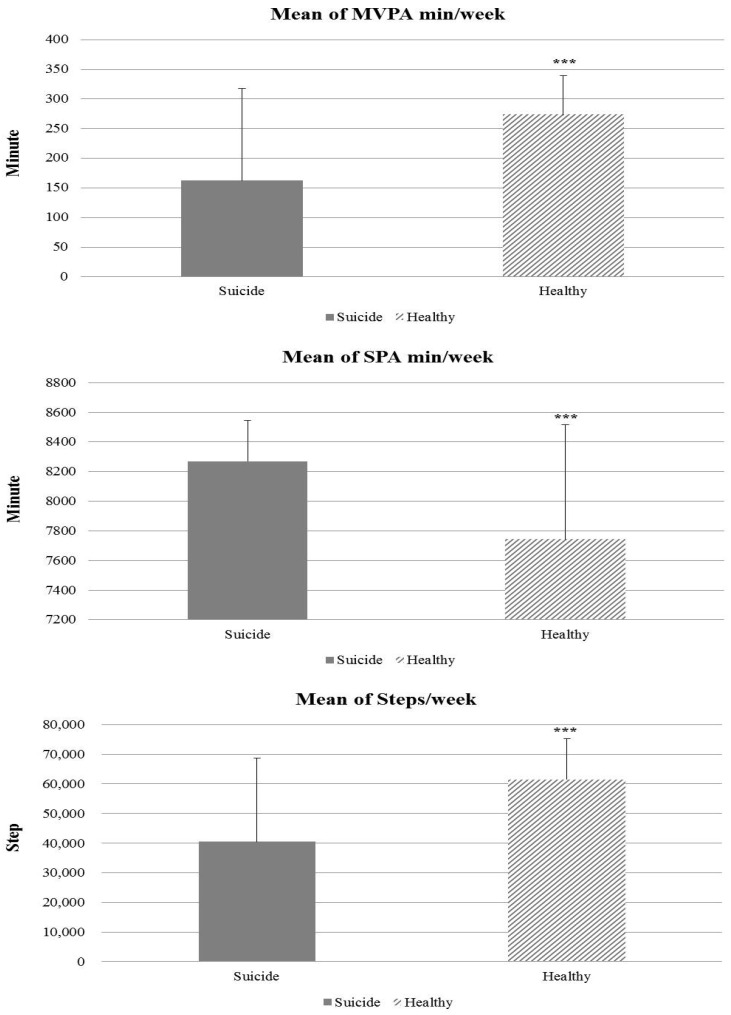
Comparison of average physical activity time and step counts calculated by an accelerometer for a week between the suicide and healthy group. The significant differences between the two groups. *** < 0.001, Error bars represent the 95% confidence interval. MVPA: moderate to vigorous physical activity, SPA: sedentary physical activity.

Figure 1 illustrates the comparison of average PA time and the number of steps using an accelerometer between two groups. The average time of participating in MVPA was 162.48 min per week in the suicide group, but the participants in the healthy group was 273.17 min per week. The results revealed a significant difference in MVPA measured by the accelerometer between the suicide and the healthy groups (*p* = 0.000; 95% CI = −155.08–−66.28). However, the average time of SPA showed the opposite result. Participants of the suicide group spent an average of 8268.66 min on sedentary behavior during a week, while the healthy group’s participants spent an average of 7743.49 min on sedentary behavior during a week (*p* = 0.000; 95% CI = 277.88–772.43). In addition, the last statistics in Figure 1 show that participants in the healthy group moved more than other group’s participants, constituting 61495.76 and 40517.34 steps, respectively (*p* = 0.000; 95% CI = −28196.42–−13759.13).

**Figure 2:**
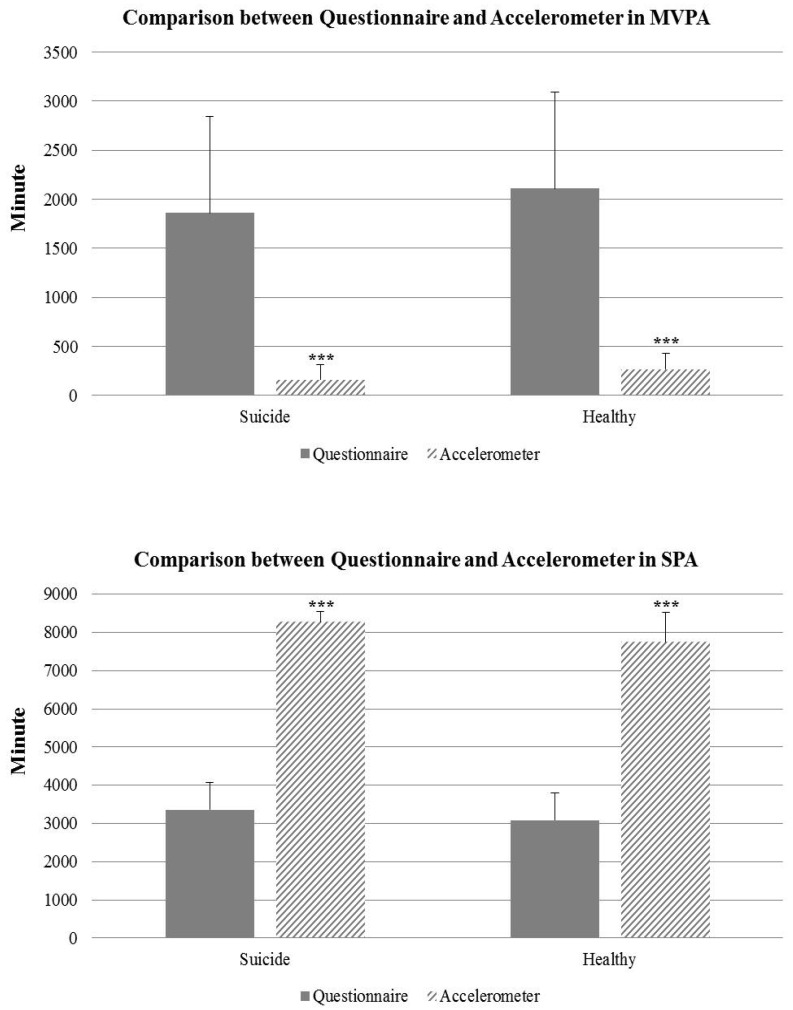
Comparison of participation time in physical activity calculated by the questionnaire and accelerometer during a week between the suicide and healthy groups. The significant differences between the two groups. *** < 0.001, Error bars represent the 95% confidence interval. MVPA: moderate to vigorous physical activity, SPA: sedentary physical activity.

Figure 2 reveals the comparison of participation time in PA calculated by the questionnaire and accelerometer for a week between the suicide and healthy groups. Participants in the suicide group responded to the questionnaire on MVPA that they had participated in physical activities for an average of 1862.42 min per week, but the average physical activity time in MVPA measured by the accelerometer was 162.48 min per week (*p* = 0.000; 95% CI = 780.75–2619.12).

Likewise, participants in the healthy group also responded to the questionnaire that the average PA time was 2109.09 min per week, but the average PA time measured by the accelerometer was only 273.17 min (*p* = 0.000; 95% CI = 1004.11–2667.72). Participants in the suicide group also responded to the questionnaire in SPA that they usually spent 3357.87 min on sedentary behavior a week, but the study found that their sedentary behavior time measured by accelerometer was 8268.65 min sitting down (*p* = 0.000; 95% CI = −7977.74–−7600.17). As with the preceding results, the healthy group’s participants also responded to the questionnaire in SPA that they spent 3086.36 min on sedentary behavior a week, but their sedentary behavior measured by the accelerometer was 7743.49 min (*p* = 0.000; 95% CI = −7446.19–−7158.97).

## 4. Discussion

Various causes provoked suicide-related behaviors such as poverty, bullying, and weak social relationships, but the suicide rate in South Korea has soared due to the severe stress caused by significant changes in a Western lifestyle based on rapid economic development [13,22]. Numerous risk factors such as old age, low economic status, and self-esteem have been introduced to date, but a lack of studies regarding the role of physical activity in suicidal behaviors may limit confirmation of its effectiveness on suicidal behaviors despite the increases in the importance of PA. Additionally, it is essential to consider that there is a possibility of inaccurate behavioral outcomes based on the types of PA assessment (i.e., questionnaire, accelerometer).

The present study investigated the association with physical activity and socioeconomic status as well as suicidal behaviors in anthropometry (i.e., age, gender, height, weight, waist circumference) matched groups and compared the differences between objective (i.e., accelerometers) and subjective (i.e., questionnaire) methods in measuring the amount of physical activity. We divided the models into four, using the data measured by accelerometers for Models 1 and 3 and by questionnaires for Models 2 and 4 to examine whether their data were related to the predictors of suicide behavior through regression analysis. The results from this study demonstrated that some variables (gender, education, occupation, and body composition) measured by accelerometer and questionnaire had no significant differences as predictors of suicidal behavior except for MVPA and SPA. However, although there were no significant differences in education levels, income, occupational clusters in accelerometers and questionnaires, the odds ratio showed that the groups with a lower education level (OR = 2.176; CI = 0.663–7.478, OR = 2.432; CI = 0.693–8.533) and lower monthly income (OR = 2.392; CI = 0.906–6.315, OR = 2.436; CI = 0.898–6.608) were more likely to have a probability for suicidal behaviors. Additionally, based on unemployment, the odds ratio demonstrated that some people who were office workers (OR = 1.459; CI = 0.448–4.752, OR = 1.358; CI = 0.413–4.461) service, and shop sales workers (OR = 1.459; CI = 0.429–2.952, OR = 1.305; CI = 0.484–3.517), and not elsewhere classified laborers (OR = 2.026; CI = 0.469–8.757, OR = 2.058; CI = 0.469–9.042) tended to attempt suicidal behaviors more than other occupational clusters. On the other hand, MVPA (*p* < 0.001; *p* < 0.001) and SPA (*p* < 0.05; *p* < 0.01) showed significant association only in Model 1 and Model 3 measured by an accelerometer. The reasons for these differences could be an underestimation of accelerometers, the overestimation of questionnaires, or a combination of both. In particular, questionnaires tend to be overestimated due to human general tendency to appear socially good (i.e., social desirability), misinterpretation of the intensity of PA, and inaccurate memory [16].

In addition, we divided those who had thought or tried suicide into suicide groups and those who had no such experience into healthy groups and used the accelerometer to accurately measure physical activity during a week. As a result, there were significant differences in the amount of physical activity between the suicide and healthy groups in MVPA, SPA, and the number of steps. On average, people in the healthy group participated in MVPA for longer than those in the suicide groups. Furthermore, on the average number of steps, we observed a considerable difference (more than 20,000 steps) between the two groups (healthy group: 61,495.76; suicide group: 40,517.34). In contrast, SPA in the suicide group, compared to the healthy group, was relatively high. This study suggests that there is a strong link to increased physical activity as one of the methods to prevent suicidal behaviors.

The finding of a strong association between PA and suicide behavior is consistent with other results in studies where PA helps individuals to have a positive mental health away from lethal suicide ideation and attempts even if intensity, frequency, and duration in PA have a low association in preventing suicide [4,23]. Furthermore, Grasdalsmoen et al. demonstrated that regular exercise was negatively associated with suicidal behaviors [24]. Therefore, to prevent depression and suicidal behaviors, we suggest that the promotion of PA is a cost-effective method and a healthy treatment in controlling depression directly linked to suicidal behavior in adults. PA is one of the preventive treatments in promoting both physical and mental health. Therefore, physical and mental healthcare professionals may prioritize the promotion of PA in their interventions [25].

The current study found that there were significant differences between the objective and subjective methods in measuring the amount of physical activity. Overall, when comparing the level of PA between the questionnaire and accelerometer, all participants in both groups indicated significantly lower MVPA measured by the accelerometer than those measured by the questionnaire. In addition, SPA measured by the accelerometer was significantly higher than those measured by the questionnaire. Although it is difficult to ignore the ease of use because measuring physical activity using questionnaires has a relatively lower cost and time, we should not ignore the excessive gap between the overestimation of accelerometers and the underestimation of questionnaires. Additionally, it should be considered that the results of prior studies and this present study showed that the measurement of physical activity through an objective measuring device such as accelerometers can provide a more accurate understanding of the associations between physical activity and suicidal behaviors. Therefore, in the course of interpreting the results of many physical activity studies, researchers will need to consider the characteristics of the measurement tools and their impact on other factors.

The present study revealed several positive strengths. First of all, the study is the first to investigate physical activity and suicidal behaviors based on age, gender, and body compositions (height, weight, and BMI) matched adults between the two groups. Moreover, this study revealed the association between physical activity and suicidal behaviors by using the data obtained by all participants wearing accelerometers for a week. This study is the first to examine the predictors of suicide behavior based on the data obtained from the accelerometer and questionnaire and revealed that differences in the predictors of suicide behavior were apparent depending on which measuring instrument was used. However, there are also a few limitations of the study. It is very difficult to assess what is the most decisive factor because suicide involves various factors. In particular, since it is more difficult to predict suicide with demographic factors such as gender, age, and educational background, it has to deeply assess risk factors based on environmental and behavioral factors in future studies. Although the fact that suicide is caused by severe depression and stress [4,23], this study has limitations that make it difficult to realize the status of depression and stress in more detail. Other studies in the future will need to demonstrate more evidence with various countries and an adequate sample size regarding the relationship between physical activity and suicidal behaviors.

## 5. Conclusions

We found that the study revealed a close association between physical activity and suicidal behaviors, and identified significant differences between the values of accelerometers and values of questionnaires to measure physical activity. Therefore, the main results of this study could be critical to systematically advance research on PA and suicidal behavior and a reference to reduce suicidal behaviors.

## Figures and Tables

**Table 1:** Characteristics and body composition for participants in the suicide and healthy group.

Variable		Suicide (*n* = 99)		Healthy (*n* = 99)	
		No. (%)	Mean ± SD	No. (%)	Mean ± SD
Gender	Male	28 (28.3)		27 (27.3)	
	Female	71 (71.7)		72 (72.7)	
Age (year)	19–29	25 (25.3)	22.36 ± 3.36	25 (25.3)	22.00 ± 2.79
	30–39	17 (17.2)	35.12 ± 2.75	18 (18.2)	35.50 ± 2.55
	40–49	18 (18.2)	44.61 ± 3.05	18 (18.2)	44.61 ± 2.72
	50–59	26 (26.3)	54.12 ± 2.99	25 (25.3)	53.68 ± 2.71
	>60	13 (13.0)	61.77 ± 1.83	13 (13.0)	61.62 ± 1.80
Anthropometrics	Height (cm)		162.12 ± 9.36		161.94 ± 7.97
	Weight (kg)		63.81 ± 12.60		63.85 ± 11.72
	Waist Circumference (cm)		80.76 ± 10.65		80.76 ± 10.29
	BMI (kg·m^−2^)		24.24 ± 4.12		24.31 ± 3.82
Education	<Elementary School	16 (16.2)		6 (6.1)	
	<Middle School	11 (11.0)		12 (12.1)	
	<High School	47 (47.5)		39 (39.4)	
	>Undergraduate	25 (25.3)		41 (41.4)	
Average Monthly Income	USD < 825	38 (38.4)		20 (20.3)	
	USD 825–1650	24 (24.2)		33 (33.3)	
	USD 1650–2476	19 (19.2)		23 (23.2)	
	USD > 2476	18 (18.2)		23 (23.2)	
Occupation	Administrators, managers, and professionals	10 (10.1)		17 (17.2)	
	Office workers	10 (10.1)		13 (13.1)	
	Service workers and shop sales workers	18 (18.2)		18 (18.3)	
	Skilled agricultural and fishery workers	3 (3.0)		3 (3.0)	
	Machine operators and assemblers	9 (9.1)		9 (9.1)	
	Laborers (not elsewhere classified)	10 (10.1)		4 (4.0)	
	Jobless (e.g., housewife, students)	39 (39.4)		34 (34.3)	

SD: standard deviation, There are no significant differences in height, weight, waist circumference, BMI between suicide and healthy group (*p* > 0.05). Education: educational status (for example, <elementary school means that people up to elementary school graduated). Total number of persons responded was 98 in the education and occupation categories in the healthy group, 1% missing from the table (did not answer), 1 USD = 1211.8 KRW (Korean Won).

**Table 2:** Multinomial logistic regression of risk factors related to suicidal behaviors between the two models.

**Variable**		**Model 1 (Accelerometer)**				**Model 2 (Questionnaire)**			
		***β***	**S.E**	**Sig.**	**OR (95% CI)**	***β***	**S.E**	**Sig.**	**OR (95% CI)**
Gender	Male				1.000				1.000
	Female	0.272	0.462	0.556	1.313 (0.531–3.249)	−0.189	0.435	0.664	0.828 (0.353–1.940)
Education	>Undergraduate				1.000				1.000
	<High School	0.494	0.408	0.227	1.638 (0.736–3.647)	0.504	0.412	0.221	1.165 (0.738–3.711)
	<Middle School	−0.068	0.620	0.913	0.934 (0.277–3.151)	0.048	0.635	0.939	1.050 (0.302–3.646)
	<Elementary School	0.777	0.630	0.217	2.176 (0.633–7.478)	0.889	0.641	0.165	2.432 (0.693–8.533)
Income	>3000 thousand KRW (USD >2476)				1.000				1.000
	2000–3000 thousand KRW (USD 1650–2476)	0.056	0.516	0.914	1.058 (0.385–2.905)	0.098	0.524	0.851	1.103 (0.395–3.081)
	1000–2000 thousand KRW (USD 825–1650)	−0.234	0.476	0.624	0.792 (0.312–2.012)	−0.22	0.482	0.664	0.800 (0.311–2.057)
	<1000 thousand KRW (USD <825)	0.872	0.495	0.078	2.392 (0.906–6.315)	0.890	0.509	0.080	2.436 (0.898–6.608)
Occupation	Jobless (e.g., housewife, students)				1.000				1.000
	Administrators and professionals	−0.751	0.514	0.144	0.472 (0.172–1.293)	−0.80	0.518	0.119	0.446 (0.161–1.231)
	Skilled agricultural and fishery workers	−0.616	0.900	0.494	0.540 (0.093–3.151)	−0.485	0.925	0.600	0.616 (0.100–3.774)
	Machine operators and assemblers	−0.187	0.645	0.772	0.829 (0.234–2.938)	0.000	0.662	1.000	1.000 (0.273–3.662)
	Office workers	0.378	0.603	0.531	1.459 (0.448–4.752)	0.306	0.607	0.615	1.358 (0.413–4.461)
	Service workers and shop sales workers	0.118	0.492	0.811	1.459 (0.429–2.952)	0.266	0.506	0.598	1.305 (0.484–3.517)
	Laborers (not elsewhere classified)	0.706	0.747	0.344	2.026 (0.469–8.757)	0.722	0.755	0.339	2.058 (0.469–9.042)
Physical Activity	Moderate to Vigorous Intensity PA	−0.005	0.002	0.001 ***	0.995 (0.992–0.998)	0.000	0.000	0.682	1. 000 (1.000–1.000)
	Sedentary Behavior	0.001	0.000	0.04 *	1.001 (1.000–1.001)	0.000	0.00	0.168	1. 000 (1.000–1.000)
		**Model 3 (Accelerometer)**				**Model 4 (Questionnaire)**			
		***β***	**S.E**	**Sig.**	**OR (95% CI)**	***β***	**S.E**	**Sig.**	**OR (95% CI)**
	Age (yr)	0.027	0.015	0.075	1.027 (0.997–1.058)	0.006	0.013	0.642	1.006 (0.981–1.032)
Characteristics	Height (cm)	0.009	0.026	0.730	0.991 (0.941–1.044)	0.005	0.025	0.849	1.005 (0.957–1.054)
	Weight (kg)	0.039	0.039	0.318	1.039 (0.963–1.122)	0.001	0.034	0.969	1.001 (0.936–1.071)
	Waist Circumference (cm)	−0.046	0.041	0.267	0.955 (0.881–1.036)	−0.004	0.037	0.906	0.996 (0.927–1.070)
Physical Activity	Moderate to Vigorous Intensity PA	−0.005	0.001	0.000 ***	0.995 (0.993–0.998)	0.000	0.000	0.655	1.000 (1.000–1.000)
	Sedentary Behavior	0.001	0.000	0.007 **	1.001 (1.000–1.001)	0.000	0.000	0.194	1.000 (1.000–1.000)

*** < 0.001, ** < 0.01, * < 0.05, S.E: Standard Error, OR: Odd Ratio, CI: Confidence Interval; Model set 1: analysis statistically adjust for gender, education, income, occupation, and physical activity measured by accelerometers. Model set 2: analysis statistically adjust for gender, education, income, occupation, and physical activity measured by questionnaires. Model set 3: analysis statistically adjust for age, height, weight, waist circumference, and physical activity measured by accelerometers. Model set 4: analysis statistically adjust for age, height, weight, waist circumference, and physical activity measured by questionnaires.

## References

[1] Naghavi M. (2019). Global, regional, and national burden of suicide mortality 1990 to 2016: Systematic analysis for the Global Burden of Disease Study 2016. BMJ.

[2] Korea Suicide Prevention Center (2020). 2020 Suicide Prevention White Book.

[3] Lee D.W., Kwon J., Yang J., Ju Y.J., Park E.-C., Jang S.-I. (2020). Suicide related indicators and trends in Korea in 2018. Health Policy Manag..

[4] Felez-Nobrega M., Haro J.M., Vancampfort D., Koyanagi A. (2020). Sex difference in the association between physical activity and suicide attempts among adolescents from 48 countries: A global perspective. J. Affect. Disord..

[5] Gutierrez P.M., Davidson C.L., Friese A.H., Forster J.E. (2016). Physical activity, suicide risk factors, and suicidal ideation in a veteran sample. Suicide Life-Threat. Behav..

[6] Brown D.R., Blanton C.J. (2002). Physical activity, sports participation, and suicidal behavior among college students. Med. Sci. Sports Exerc..

[7] Owiti J., Bhui K.S. (2012). The reciprocal relationship between physical activity and depression in older European adults. Évid. Based Nurs..

[8] Rothon C., Edwards P., Bhui K., Viner R.M., Taylor S., Stansfeld S.A. (2010). Physical activity and depressive symptoms in adolescents: A prospective study. BMC Med..

[9] Simon T.R., Powell K.E., Swann A.C. (2004). Involvement in physical activity and risk for nearly lethal suicide attempts. Am. J. Prev. Med..

[10] Brosnahan J., Steffen L.M., Lytle L., Patterson J., Boostrom A. (2004). The relation between physical activity and mental health among Hispanic and non-Hispanic white adolescents. Arch. Pediatr. Adolesc. Med..

[11] Lee S.W., Shim J.S., Song B.M., Lee H.J., Bae H.Y., Park J.H., Choi H.R., Yang J.W., Heo J.E., Cho S.M.J. (2018). Comparison of self-reported and accelerometer-assessed measurements of physical activity according to socio-demographic characteristics in Korean adults. Epidemiol. Health.

[12] Cho K.O. (2014). Physical activity and suicide attempt of South Korean adolescents–Evidence from the eight Korea youth risk behaviors web-based survey. J. Sports Sci. Med..

[13] Kwon M., Lee J. (2017). Physical activity and suicidal thoughts in male and female adolescents. J. Korean Soc. Sch. Health.

[14] Skender S., Ose J., Chang-Claude J., Paskow M., Brühmann B., Siegel E.M., Steindorf K., Ulrich C.M. (2016). Accelerometry and physical activity questionnaires—A systematic review. BMC Public Health.

[15] Sallis J.F., Saelens B.E. (2000). Assessment of physical activity by self-report: Status, limitations, and future directions. Res. Q. Exerc. Sport.

[16] Atienza A.A., Moser R.P., Perna F., Dodd K., Ballard-Barbash R., Troiano R.P., Berrigan D. (2011). Self-reported and objectively measured activity related to biomarkers using NHANES. Med. Sci. Sports Exerc..

[17] Tucker J.M., Welk G.J., Beyler N.K., Kim Y. (2016). Associations between physical activity and metabolic syndrome: Comparison between self-report and accelerometry. Am. J. Health Promot. AJHP.

[18] Troiano R.P., Berrigan D., Dodd K.W., Mâsse L.C., Tilert T., McDowell M. (2008). Physical activity in the United States measured by accelerometer. Med. Sci. Sports Exerc..

[19] Faul F., Erdfelder E., Buchner A., Lang A.G. (2009). Statistical power analyses using G*Power 3.1: Tests for correlation and regression analyses. Behav. Res. Methods.

[20] Lim J., Sung H., Lee O., Kim Y. (2020). Physical activity in South Korea measured by accelerometer: The Korea National Health and Nutrition Examination Survey VI 2014–2015. Korean J. Sport Sci..

[21] Lee C.G., Cho Y., Yoo S. (2013). The relations of suicidal ideation and attempts with physical activity among Korean adolescents. J. Phys. Act. Health.

[22] Michael S.L., Lowry R., Merlo C., Cooper A.C., Hyde E.T., McKeon R. (2020). Physical activity, sedentary, and dietary behaviors associated with indicators of mental health and suicide risk. Prev. Med. Rep..

[23] Grasdalsmoen M., Eriksen H.R., Lønning K.J., Sivertsen B. (2020). Physical exercise, mental health problems, and suicide attempts in university students. BMC Psychiatry..

[24] Paffenbarger R.S., Lee I.M., Leung R. (1994). Physical activity and personal characteristics associated with depression and suicide in American college men. Acta Psychiatr. Scand..

[25] Department of Health (2011). No Health without Mental Health: A Cross-Government Mental Health Outcomes Strategy for People of All Ages. Supporting Document-the Economic Case for Improving Efficiency and Quality in Mental Health.

